# Impact of intravenous immunoglobulin on the dopaminergic system and immune response in the acute MPTP mouse model of Parkinson’s disease

**DOI:** 10.1186/1742-2094-9-234

**Published:** 2012-10-09

**Authors:** Isabelle St-Amour, Mélanie Bousquet, Isabelle Paré, Janelle Drouin-Ouellet, Francesca Cicchetti, Renée Bazin, Frédéric Calon

**Affiliations:** 1 Centre de Recherche du CHUL (CHUQ), Axe Neurosciences, T2-05, 2705, boulevard Laurier, Québec, QC, Canada , G1V 4G2; 2 Faculté de Pharmacie, Université Laval, Québec, QC, Canada , G1V 0A6; 3 Département de Recherche et Développement, Héma-Québec, Québec, QC, Canada , G1V 5C3; 4 Département de Psychiatrie & Neurosciences, Faculté de Médecine, Université Laval, Québec, QC, Canada, G1V 0A6

**Keywords:** Intravenous immunoglobulin, Parkinson’s disease, Immunity, Neurodegeneration, MPTP, Dopamine

## Abstract

Intravenous immunoglobulin (IVIg) is a blood-derived product, used for the treatment of immunodeficiency and autoimmune diseases. Since a range of immunotherapies have recently been proposed as a therapeutic strategy for Parkinson’s disease (PD), we investigated the effects of an IVIg treatment in a neurotoxin-induced animal model of PD. Mice received four injections of MPTP (15 mg/kg) at 2-hour intervals followed by a 14-day IVIg treatment, which induced key immune-related changes such as increased regulatory T-cell population and decreased CD4^+^/CD8^+^ ratio. The MPTP treatment induced significant 80% and 84% decreases of striatal dopamine concentrations (*P* < 0.01), as well as 33% and 40% reductions in the number of nigral dopaminergic neurons (*P* < 0.001) in controls and IVIg-treated mice, respectively. Two-way analyses of variance further revealed lower striatal tyrosine hydroxylase protein levels, striatal homovanillic acid concentrations and nigral dopaminergic neurons (*P* < 0.05) in IVIg-treated animals. Collectively, our results fail to support a neurorestorative effect of IVIg on the nigrostriatal system in the MPTP-treated mice and even suggest a trend toward a detrimental effect of IVIg on the dopaminergic system. These preclinical data underscore the need to proceed with caution before initiating clinical trials of IVIg in PD patients.

## Background

Central and peripheral activation of the innate and adaptive immune systems have been associated with neurodegenerative diseases such as Parkinson’s disease (PD). Elevated concentrations of cytokines, such as TNFα, IL-1β, IL-6, TGFβ (transforming growth factor-beta) and IFNγ have been reported in mouse models of dopaminergic (DAergic) denervation [1,2] as well as in the substantia nigra pars compacta (SNpc) and striatum of PD patients [2-6]. Similarly, increased concentrations of macrophage migration inhibition factor, IL-2, IL-6, TNFα and TNFα receptor-1 have been measured in the blood of PD patients [7-13]. Migration of both CD4^+^ (helper) and CD8^+^ (cytotoxic) T-lymphocytes have been identified within the SNpc of PD patients and further associated with nigrostriatal denervation in a mouse model of PD through a CD4^+^ T-cell-dependent Fas/Fas ligand (FasL) cytotoxic pathway [14]. Immunological abnormalities observed in PD patients and animal models suggest an overall disruption of the immune system in the disease, but their causal role is still highly debated. It remains to be demonstrated whether immunological abnormalities are relevant therapeutic targets for neurodegenerative disorders.

Recently, intravenous immunoglobulin (IVIg) has been proposed in the treatment of neurodegenerative diseases. The safety and tolerability profiles of IVIg have justified the initiation of phase II and phase III clinical trials in Alzheimer’s disease (AD) patients and in individuals suffering from mild cognitive impairments (reviewed in [15]). IVIg is a therapeutic preparation of over 98% human IgG purified from the plasma of thousands of healthy donors [16-19]. Besides its routine use for a growing number of autoimmune diseases, beneficial effects of IVIg have also been reported in immune-mediated neurological diseases such as chronic inflammatory demyelinating polyneuropathy, Guillain–Barré syndrome, multiple sclerosis and multifocal motor neuropathy (reviewed in [20-23]).

Although the mechanisms of action of IVIg remain only partially understood, anti-inflammatory and immunomodulatory effects have been described both *in vitro* and *in vivo*[24-28]. In theory, some of these functions could correct key immunologic defects described in PD. For example, in humans, IVIg treatments decrease plasma levels of TGFβ, IL-1β, IL-6, IL-8, IFNγ and TNFα [29,30], all shown to be upregulated in PD. *In vitro,* IVIg has also been reported to decrease phagocytosis in microglia, to modulate transendothelial cell migration, adhesion and rolling, and to interfere with the Fas/FasL cytotoxic pathway [31-34]. More recently, increased hippocampal neurogenesis following IVIg treatment has been reported in a mouse model of AD [35]. Furthermore, active and passive immunization against α-synuclein (α-syn), the main component of the neuronal cytoplasmic inclusions found in PD [36,37], has been shown to reduce neuropathology and behavioral deficits in an α-syn transgenic mouse model [38,39]. In line with these latter observations, antibodies specific to α-syn have recently been isolated from IVIg [40], further suggesting a potential clinical application for the use of IVIg to achieve passive immunization in PD.

Current therapies in PD are mainly symptomatic, and no drugs have ever obtained a label of disease modification or neuroprotection from health agencies [41,42]. In light of the existing data for the benefits of IVIg in autoimmune and neurological diseases, we undertook to investigate whether such an approach could also benefit PD patients. To test this hypothesis, we evaluated whether IVIg could lead to the neurorestoration of the DAergic system after a nigrostriatal lesion. We used a post-MPTP paradigm where the IVIg treatment was delivered after the MPTP insult. This approach avoids unwanted interference of IVIg with MPTP toxicokinetics and is more representative of the typical clinical setting where the treatment is administered after the diagnosis [43].

## Materials and methods

### Reagents

All biochemical reagents were purchased from J.T. Baker (Phillipsburg, NJ, USA) unless otherwise specified.

### Animals, MPTP administration and IVIg treatment

Eight-week-old C57BL6J males (22 to 27 g), purchased from Charles River Laboratories (Montréal, QC, Canada) were housed three per cage with free access to food and water. All procedures were approved by the Animal Research Committee of Laval University.

**Figure 1:**
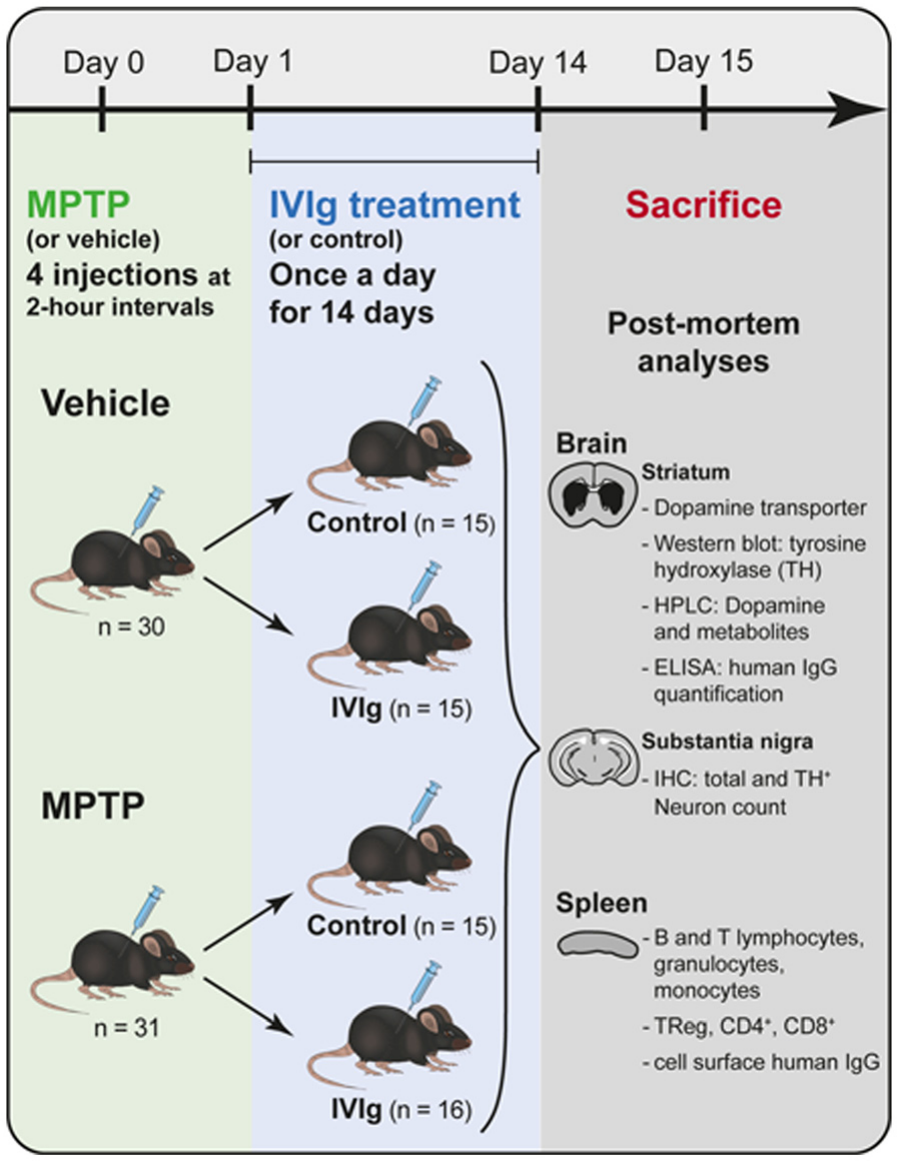
**Experimental design.** Mice received four injections of the neurotoxin MPTP (15 mg/kg; MPTP group) or an equivalent volume of saline (vehicle group) every 2 hours on day 0. Twenty hours after the last injection, mice received intraperitoneal injections of 30 mg intravenous immunoglobulin (IVIg) or an equivalent volume of 0.2 M glycine pH 4.0 (control group) on the first day (bolus) and 10 mg daily (maintenance dose) for the remaining 13 days. Mice were sacrificed on day 15 by intracardiac perfusion under deep anesthesia.

Animals were injected intraperitoneally with MPTP neurotoxin following a standard acute protocol [43-45] and were sacrificed 14 days later (Figure 1). On day 0, the mice received four injections of an MPTP–HCl solution (15 mg free base/kg; Sigma-Aldrich, Oakville, ON, Canada) freshly dissolved in 0.9% saline, at 2-hour intervals. To avoid that the pharmacologic intervention under study alters MPTP toxicokinetics, Jackson-Lewis and Przedborski suggested delaying the beginning of the treatment for at least 8 hours after the last MPTP injection [43]. An IVIg treatment posology of 0.4 g × kg^-1^ × week^-1^ has shown efficacy in a recent AD clinical trial [46]. However, the mouse metabolism is faster than that of humans, as exemplified by the IVIg half-life of 89 hours in mice (unpublished data) instead of 35 days in humans [17]. To match the human dosage as closely as possible, we thus selected a dose of 0.4 g × kg^-1^ × day^-1^. To quickly reach therapeutic concentrations, mice (*n* = 15 or 16/group) received a bolus dose of 30 mg IVIg (~1.2 g/kg from a 100 mg/ml human IgG solution, diluted in 0.2 M glycine pH 4.25 – Gammunex™; Grifols, Mississauga, ON, Canada) or an equivalent volume of glycine (0.2 M, pH 4.25) 20 hours following the last MPTP injection. For the remaining 13 days, animals were injected daily with 10 mg/day (~0.4 g × kg^-1^ × day^-1^) IVIg or glycine (maintenance dose) for a total treatment duration of 14 days. The animals were sacrificed 2 weeks after the last MPTP injection to probe for a neurorestorative effect of IVIg on the ongoing MPTP-induced neurodegeneration of the DAergic system.

### Tissue preparation for postmortem analyses

Terminal intracardiac perfusion was performed under deep anesthesia (ketamine/xylazine). After transcardiac administration of 50 ml PBS buffer (Bioshop Canada Inc., Burlington, ON, Canada) containing protease and phosphatase inhibitors (SigmaFAST Protease Inhibitor Tablets (Sigma-Aldrich) with 50 mM sodium fluoride and 1 mM sodium pyrophosphate), both spleen and brain were collected. Brain hemispheres were separated: the striatum was dissected from the rostral section of the right hemisphere, snap-frozen on dry ice and stored at −80°C. The caudal section was post-fixed in 4% paraformaldehyde pH 7.4 and sliced with a freezing microtome (coronal brain sections of 25 μm). The left hemisphere was snap-frozen in 2-methyl-butane and stored at −80°C for cryostat coronal brain sections (12 μm). Splenocytes were recovered from the spleen following mechanical disaggregation, frozen at −80°C in a cryoprotective medium (Iscove’s Modified Dulbecco’s Medium supplemented with 20% fetal bovine serum and 5% dimethylsulfoxide) and kept in liquid nitrogen until further use.

### Spleen analyses

#### Flow cytometry

Cell surface marker expression on splenocytes were analyzed using AF647-conjugated anti-CD3 (clone 17A2; BD Biosciences, Mississauga, ON, Canada), eFluor 780-conjugated anti-B220 (clone RM4-5; BD Biosciences), PE-Cy7-conjugated anti-CD4 (clone GK1.5; eBioscience, San Diego, CA, USA), eFluor 450-conjugated anti-CD8a (clone 53–6.7; eBioscience), APC-conjugated anti-CD11b (clone M1/70; BD Biosciences), FITC-conjugated anti-Gr1 (LY-6 G clone RB6-8C5; eBioscience) or relevant isotypic controls in PBS–1% BSA. For human IgG detection, splenocytes were labeled with PE-conjugated anti-CD45 and FITC-conjugated anti-human IgG. For regulatory T-lymphocyte analyses, splenocytes were stained on ice for 30 minutes with PE-conjugated anti-CD25 (clone PC61.5; eBioscience) and APC-conjugated anti-CD4 (clone RM4-5; BD Bioscience) followed by permeabilization and fixation using the Foxp3 staining buffer (eBioscience) and staining with eFluor450-conjugated anti-Foxp3 (clone FJK-16s; eBioscience) following the manufacturer’s instructions. Cells were acquired and analyzed using a CyFlow ML (Partec, Swedesboro, NJ, USA) cytometer and FCS express software (De Novo Software, Los Angeles, CA, USA).

#### ELISPOT analyses

To determine whether the injection of IVIg triggered an anti-human IgG immune response, an ELISPOT test was performed. Briefly, splenocytes were unfrozen, washed, counted, plated on human IgG-coated wells (Multiscreen® HTS filter plate; Millipore Corporation, Billerica, MA, USA) blocked with 5% fetal bovine serum and left immobile for 16 hours at 37°C, 10% CO_2_ for antibody secretion. After washing the cells, anti-human specific mouse immunoglobulins were detected using a horseradish peroxidase-conjugated anti-mouse IgG (heavy and light chain specific; Jackson ImmunoResearch Laboratories Inc., West Grove, PA, USA) and TrueBlue™ Peroxydase Substrate (KPL, Mandel Scientific, Gaithersburg, MD, USA). Each spot was counted under a dissection microscope and considered a single anti-human specific B cell. Results from mice treated with IVIg were compared with controls.

### Brain analyses

#### Striatum

##### ELISA and Western immunoblot analyses

Each striatum was homogenized in 8 volumes of lysis buffer per milligram of tissue (150 mM NaCl, 10 mM Na_2_HPO_4_, 0.5% sodium dodecylsulfate, 0.5% sodium deoxycholate, 1% Triton X-100 containing Complete™ protease inhibitors cocktail (Roche, Indianapolis, IN, USA), 10 μg/ml pepstatin A and 1 mM each of sodium fluoride and sodium orthovanadate as phosphatase inhibitors) and was sonicated three times for five 1-second pulses. The solution was centrifuged at 100,000×*g* for 20 minutes at 4°C, and the supernatant was retrieved and kept at −80°C for ELISA and immunoblotting. The protein concentration was determined using a bicinchoninic acid assay (Pierce, Rockford, IL, USA).

An ELISA specific to human IgG was utilized to determine the striatal concentration of IVIg using goat anti-human IgG Fc-specific antibodies (Jackson ImmunoResearch Laboratories Inc.). For immunoblot analyses, proteins (20 μg/samples) were heated at 95°C for 5 minutes in Laemmli’s loading buffer and separated by SDS-PAGE on a 10% polyacryamide gel, before transferring to a polyvinylidene fluoride membrane (Immobilon-P™; Millipore Corporation) that was blocked in 5% nonfat dry milk, 0.5% BSA, 0.1% Tween 20 in PBS buffer as previously described [47]. Tyrosine hydroxylase (TH) protein was detected using rabbit anti-TH (1:5,000, #P40101; Pel-Freez, Rogers, AR, USA) primary antibody followed by horseradish peroxidase-labeled secondary antibody and chemiluminescence reagents (Lumiglo Reserve; KPL) as previously described [48]. Membranes were also probed for β-actin (1:10,000; Applied Biological Materials Inc., Richmond, BC, Canada) as a control for protein load. Band intensities were quantified using a KODAK Imaging Station 4000 MM Digital Imaging System (Molecular Imaging Software version 4.0.5f7; Carestream Health, Rochester, NY, USA).

##### Catecholamine and indolamine quantification

Ten slices of 20 μm rostral striata were homogenized in 200 μl of 0.1 N perchloric acid (Mallinckrodt Baker, Phillipsburg, NJ, USA) and centrifuged at 12,000×*g* for 10 minutes at 4°C. HPLC with electrochemical detection was used to evaluate the concentration of dopamine (DA), 3,4-dihydroxyphenylacetic acid (DOPAC) and homovanillic acid (HVA) in striatal supernatant, as previously described [48]. Briefly, 50 μl supernatant were injected into the chromatograph consisting of a Waters 717 plus autosampler automatic injector, a Waters 1525 binary pump equipped with an Atlantis dC18 column, a Waters 2465 electrochemical detector, and a glassy carbon electrode (Waters Ltd, Lachine, QC, Canada). The electrochemical detector was set at 10 nA. The mobile phase consisted of 47.8 mM NaH_2_PO_4_, 0.9 m M sodium octyl sulfate, 0.4 mM ethylenediamine tetraacetic acid, 2 mM NaCl and 8% (v/v) methanol at pH 2.9 and was delivered at 0.8 ml/minute. Peaks were identified using Breeze Software (Waters Ltd) and HPLC quantifications were normalized to protein concentrations.

#### Dopamine transporter quantification

DA transporter (DAT) was evaluated with 3β-(4-^125^I-iodophenyl) tropane-2-carboxylic acid isopropylester (^125^I-RTI-121, 2,200 Ci/mmol; NEN-DuPont, Boston, MA, USA), as previously described [45,49]. Slide-mounted brain sections were preincubated at room temperature for 30 minutes in phosphate buffer pH 7.4 followed by a 90-minute incubation with 20 pM ^125^I-RTI-121. Nonspecific binding was determined in the presence of 0.1 μM mazindol (Novartis Pharmaceuticals, Dorval, QC, Canada). Sections were then washed with phosphate buffer followed by distilled water, dried overnight and exposed to Kodak BioMax film for 16 hours (Sigma-Aldrich). Densitometry was quantified using the ImageJ Analysis Software (National Institutes of Health, USA). The average labeling for each area was calculated from the mean of six adjacent brain sections from the rostral striatum of a 1/10 series of the same animal.

#### Substantia nigra

##### Immunohistochemistry

To visualize TH-positive neurons of the SNpc, sections were first incubated for 30 minutes in 3% H_2_O_2_ and blocked with 5% normal goat serum and 0.1% Triton in PBS for 30 minutes. After an overnight incubation with an anti-TH antibody (1:5,000; Pel-Freez), sections were washed three times in PBS and incubated for 1 hour with biotin-conjugated anti-rabbit antibody. After further washing, the sections were placed in a solution containing ABC (Elite kit; Vector Laboratories, Burlington, ON, Canada) for 1 hour at room temperature. The bound peroxidase was revealed with 0.5 mg/ml DAB (Sigma-Aldrich) and 0.01% hydrogen peroxide in 0.05 M Tris (pH 7.6). The reaction was stopped by extensively washing the sections in PBS. The sections were counterstained with cresyl violet, dehydrated and cover slipped. Photomicrographs were taken with a Microfire 1.0 camera (Optronics, Goleta, CA, USA) linked to an E800 Nikon 274 microscope (Nikon Inc., Québec, QC, Canada) using the imaging software Picture Frame.

##### Stereological counts of TH-positive and cresyl-violet-stained cells

The total number of TH-positive and TH-negative neurons of the SNpc was quantified stereologically on seven sections of a 1/5 series, as previously described [48-50]. Selected sections, at intervals of 125 μm, were counted in a blinded fashion by two independent investigators using Stereo Investigator software (MicroBrightfield, Williston, VT, USA) integrated to an E800 Nikon 274 microscope. After delineating the SNpc at low magnification (4× objective) a point grid was overlaid onto each section. Stained cells (TH-positive or cresyl-violet stained) with a clearly defined and intact nucleus were counted using the optical fractionator method at higher magnification (20× objective). The counting variables were as follows: distance between counting frames, 150 μm×150 μm; counting frame size, 75 μm; guard zone thickness, 1 μm. Cells were counted only if they did not intersect forbidden lines.

### Statistical analyses

Statistical analyses were performed using the JMP software (version 9.0.2; SAS Institute Inc., Cary, IL, USA) and Prism 4.0c (GraphPad Software Inc., La Jolla, CA, USA). A Bartlett test was first performed on all data to verify equal variance. In cases of equal variance, statistical differences were determined using one-way analysis of variance (ANOVA) followed by post-hoc test (Tukey’s or Dunnett’s) for comparison between groups. When variances were unequal, a Welch ANOVA followed with Dunnett’s multiple comparison test was employed. If a Gaussian distribution could not be assumed, the Kruskal–Wallis nonparametric test was used followed by Dunn's post-test. The survival curves were compared using the log-rank test. To evaluate the overall effects of IVIg in both the vehicle and MPTP groups, two-way ANOVAs were also performed.

## Results

### Health status and IVIg treatment

**Figure 2:**
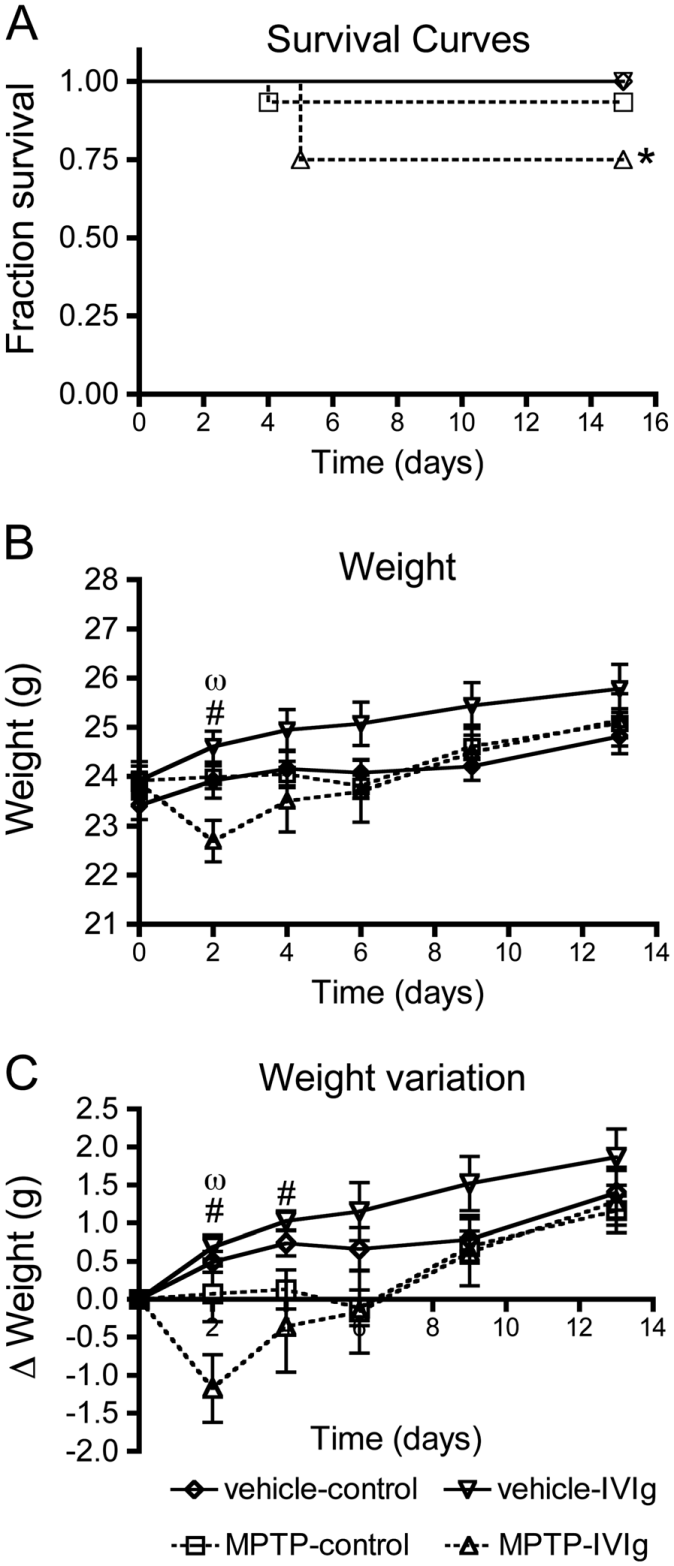
**Intravenous immunoglobulin injection increases MPTP-related weight loss and lethality.** Lethality and weight loss rate were monitored throughout the experiment. (**A**) Mortality rate induced by MPTP treatment was higher in the intravenous immunoglobulin (IVIg) group. (**B**) Weight and (**C**) weight variation of the animals excluding all the mice that died before the end of the protocol. Data are presented as mean ± standard error of the mean of 12 to 15 animals. Statistical analysis: (**A**) log-rank test was performed to compare survival curves, *P <0.05 MPTP-IVIg versus vehicle groups. (**B**),(**C**) One-way analysis of variance followed by Tukey’s multiple comparison test, ^#^*P* <0.05 MPTP-IVIg versus vehicle-IVIg group, ^ω^*P* <0.05 MPTP-IVIg versus MPTP-control group.

Weight loss and mortality is commonly observed in the acute MPTP protocol, particularly shortly after injections of the toxin [43]. In this study, MPTP administration led to the death of 5/31 mice between days 4 and 5 after toxin injection (Figure 2A): one mouse in the MPTP-control group (*n =* 15) and four of the MPTP-IVIg-treated animals (*n* = 16). MPTP-induced lethality was thus significantly higher in the MPTP-IVIg group. These animals were excluded from all postmortem analyses. An increase in weight loss, paralleling the mortality rate, was also observed in surviving animals from the MPTP-IVIg group (Figure 2B,C), specifically at day 2. Despite the fact that IVIg alone did not lead to increased lethality or weight loss in the vehicle group, the treatment tended to potentiate the detrimental effect of MPTP and thus impact the general health status of the treated animals.

### IVIg administration increases the proportion of the regulatory T cells in the spleen

**Figure 3:**
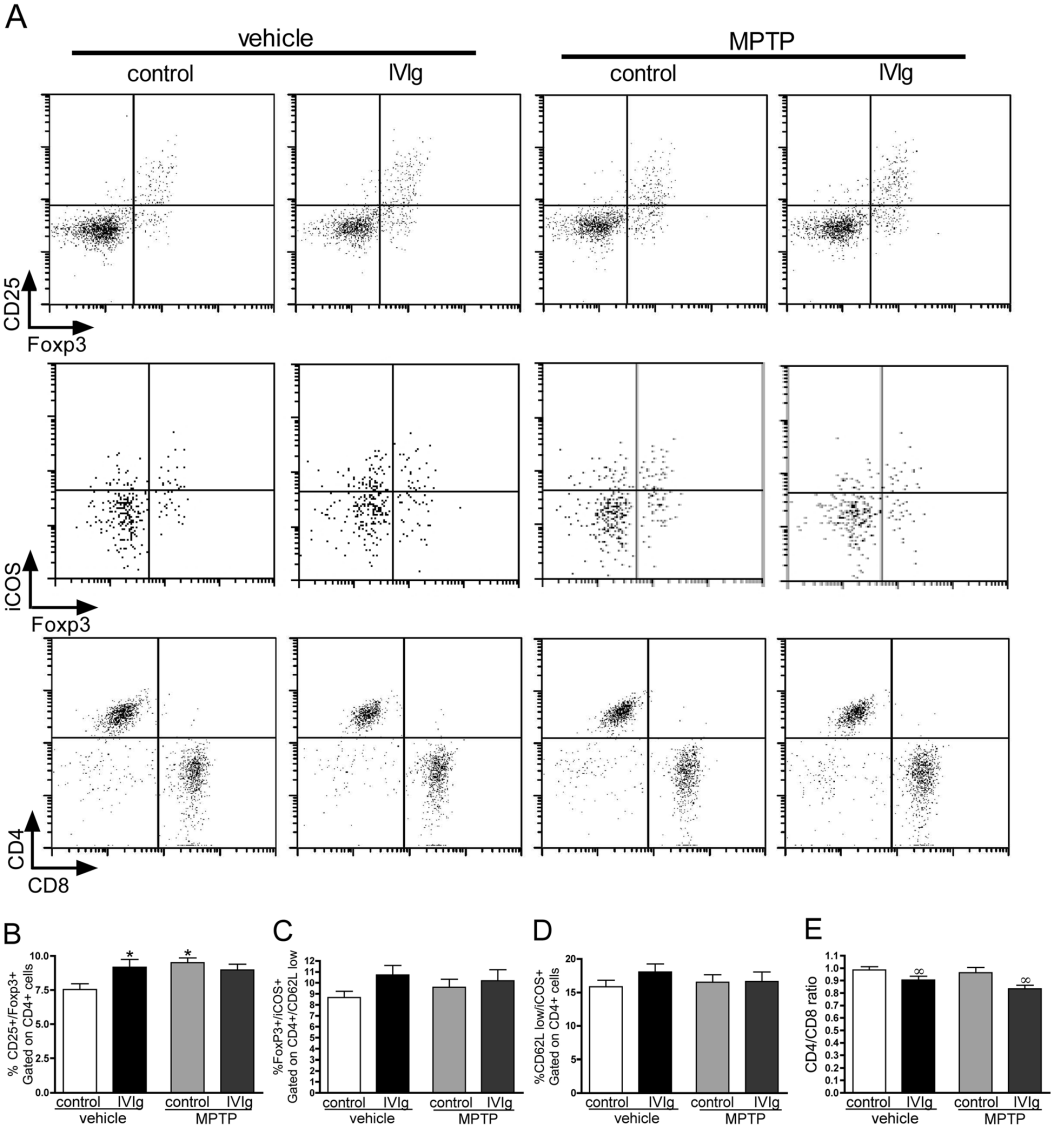
**Intravenous immunoglobulin injection modulates the relative populations of T lymphocytes.** Flow cytometry analyses on splenocytes labeled for regulatory (CD4^+^, CD25^+^ and Foxp3^+^) iCOS^+^CD4^+^ (CD62L low, CD4^+^, iCOS^+^ and Foxp3^+^), helper (CD4^+^) and cytotoxic (CD8^+^) T cells. (**A**) Representative plots showing regulatory T cell (Treg; upper panels, gated on CD4^+^ cells) iCOS^+^CD4^+^ (middle panels, gated on CD4^+^/CD62L low cells) and CD4^+^/CD8^+^ (lower panels, gated on CD3^+^ cells) analyses. (**B**) Intravenous immunoglobulin (IVIg) induced a mild but significant increase in Treg relative population but not in (**C**) iCOS^+^/Foxp3^+^ or (**D**) iCOS^+^ populations. IVIg treatment also decreased the (**E**) CD4^+^/CD8^+^ cell ratio. (**B**) to (**E**) Results are presented as mean ± standard error of the mean (10 to 15 animals). Statistical analysis: *P <0.05 versus vehicle-control group, one-way analysis of variance (ANOVA), followed by Dunnett’s multiple comparison test, ^∞^*P* <0.05 IVIg versus control, two-way ANOVA.

We next evaluated the effects of IVIg on the splenic population of regulatory T cells (Tregs), which are known to be upregulated by IVIg in patients and mouse models of inflammatory disorders [51-54]. Indeed, Tregs suppress immune activation and maintain immune homeostasis and tolerance [55,56], while protecting nigrostriatal afferents in an MPTP mouse model of PD [57-59]. Our data revealed that repeated injections of IVIg resulted in an increase in the percentage of splenic Tregs. A 21.6% increase of CD25^+^/Foxp3^+^ Tregs in the CD4^+^ population following IVIg treatment was observed, but only in the vehicle group (Figure 3A,B; 7.55% vs. 9.17% CD25^+^/Foxp3^+^ gated on CD4^+^, vehicle-control and vehicle-IVIg respectively). MPTP treatment also induced a 26.1% increase in the relative Treg population (Figure 3A,B; MPTP-control: 9.52% Treg), but the IVIg treatment did not further increase the Treg population in the MPTP group. We also evaluated the sub-population of iCOS^+^CD4^+^ T cells (Figure 3), as they have previously been associated with IVIg-induced regulation of the central nervous system (CNS) inflammatory response [54], but no significant effects were observed in any of the treated groups. Granulocyte, monocyte, B-lymphocyte and T-lymphocyte populations in the splenocytes of all animals were also analyzed but no statistical differences were observed between groups (data not shown). However, IVIg injections also led to a significant decrease in the CD4^+^/CD8^+^ T lymphocytes ratio in the vehicle and MPTP groups compared with controls, respectively (Figure 3).

### Bioavailability of IVIg in brain and periphery

**Figure 4:**
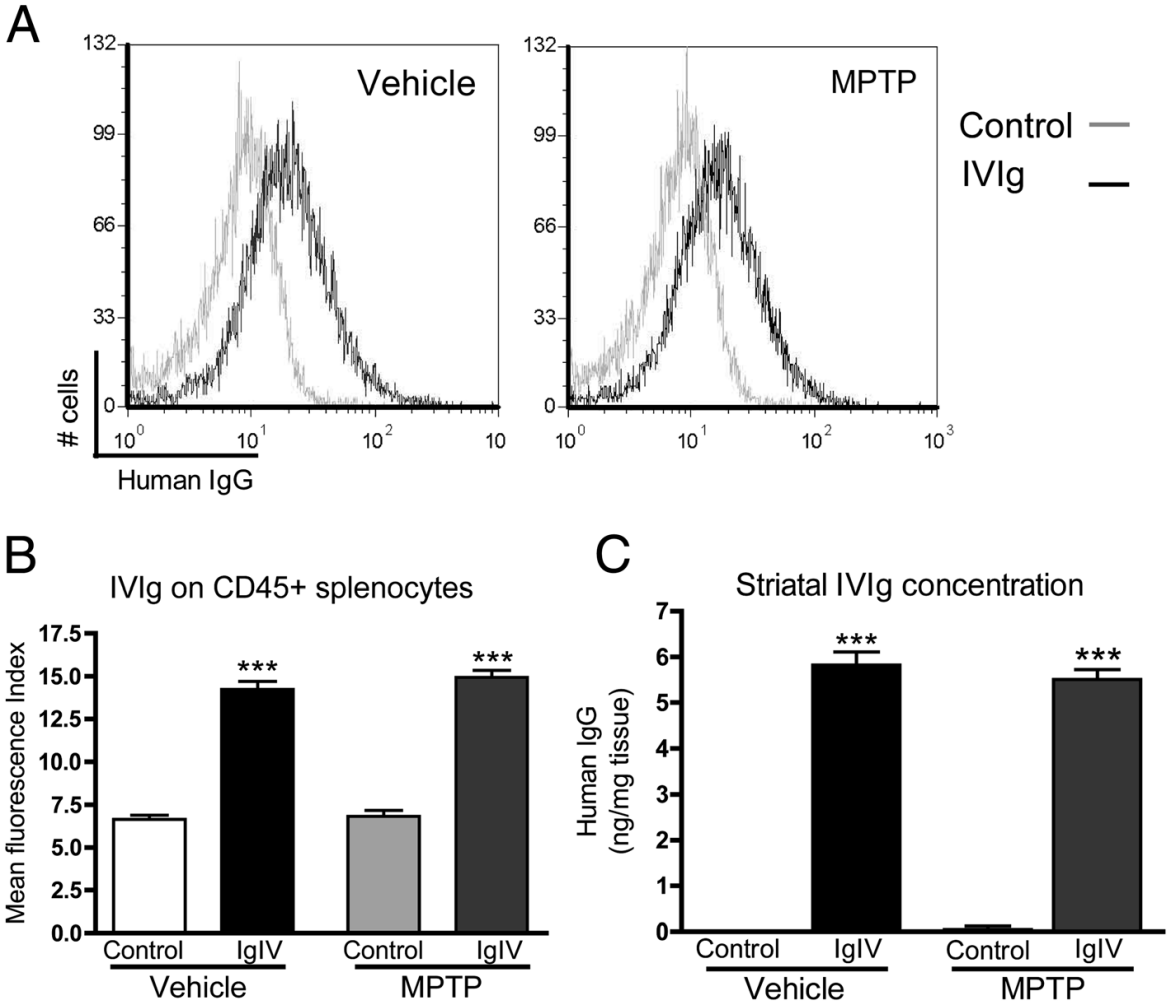
**Detectable amounts of intravenous immunoglobulin in the brain and splenocytes of treated mice.** Human IgG concentrations were evaluated on splenocytes and in the striatum of intravenous immunoglobulin (IVIg) and control mice. (**A**) Representative flow cytometric analyses and (**B**) mean fluorescence intensity (MFI) quantification of extracellular human IgG on CD45^+^ splenocytes. Note that the MFI of the control groups is equivalent to isotypic control and autofluorescence (data not shown). (**C**) Concentrations of IVIg were measured in striatum homogenates from intracardially perfused mice using a specific ELISA. (**B**),(**C**) Data presented as mean ± standard error of the mean of 12 to 15 animals, ***P <0.001 versus control groups, one-way analysis of variance followed by Tukey’s multiple comparison test.

We further measured the bioavailability of IVIg in the brain and spleen of IVIg-treated mice. Detectable amounts of extracellular IVIg were present on splenic CD45^+^ leukocytes as evaluated by flow cytometry (Figure 4A,B) with a mean fluorescence intensity of 14.54 ± 0.32 in the IVIg groups versus a baseline autofluorescence intensity of 6.72 ± 0.21 for controls. Using a human specific anti-IgG ELISA, we also determined that the concentrations of IVIg in striatal homogenates were as high as 5.8 ± 0.2 and 5.5 ± 0.3 ng IVIg/mg tissue in the vehicle-IVIg and MPTP-IVIg groups, respectively (Figure 4C). These data suggest that detectable amounts of human IgG are present in the brain and on the surface of circulating leukocytes after a 14-day treatment with IVIg in this parkinsonian mouse model.

### IVIg induces a minimal immune response

To determine whether repeated IVIg injections induced an anti-human IgG specific adaptive immune response, ELISPOT analyses were performed (*n* = 10 per group). With this technique, we were able to assess the number of splenocytes secreting a specific anti-human IgG antibody in control and IVIg groups, 14 days post MPTP. Although there was a significant increase in the number of splenic cells reactive to human IgG following IVIg administration (two-way ANOVA, *P* < 0.05), it remained very low. The amount of anti-human IgG specific cells was below 4/100,000 splenocytes in all animals. The absolute number of antibody secreting cells per spleen was 445 ± 173 and 154 ± 98 for the vehicle-IVIg and MPTP-IVIg group (mean ± standard error of the mean) versus 34 ± 22 and 23 ± 12 for the vehicle-control and MPTP-control groups. For comparison purposes, in mouse models of autoimmune diseases, the reported absolute number of antibody secreting cells to myeloperoxidase, nucleolin and dsDNA are higher than 11,000, 17,000 and 33,000 specific cells/spleen, respectively [60,61].

### Effects of MPTP and IVIg on the striatal components of the dopaminergic system

**Figure 5:**
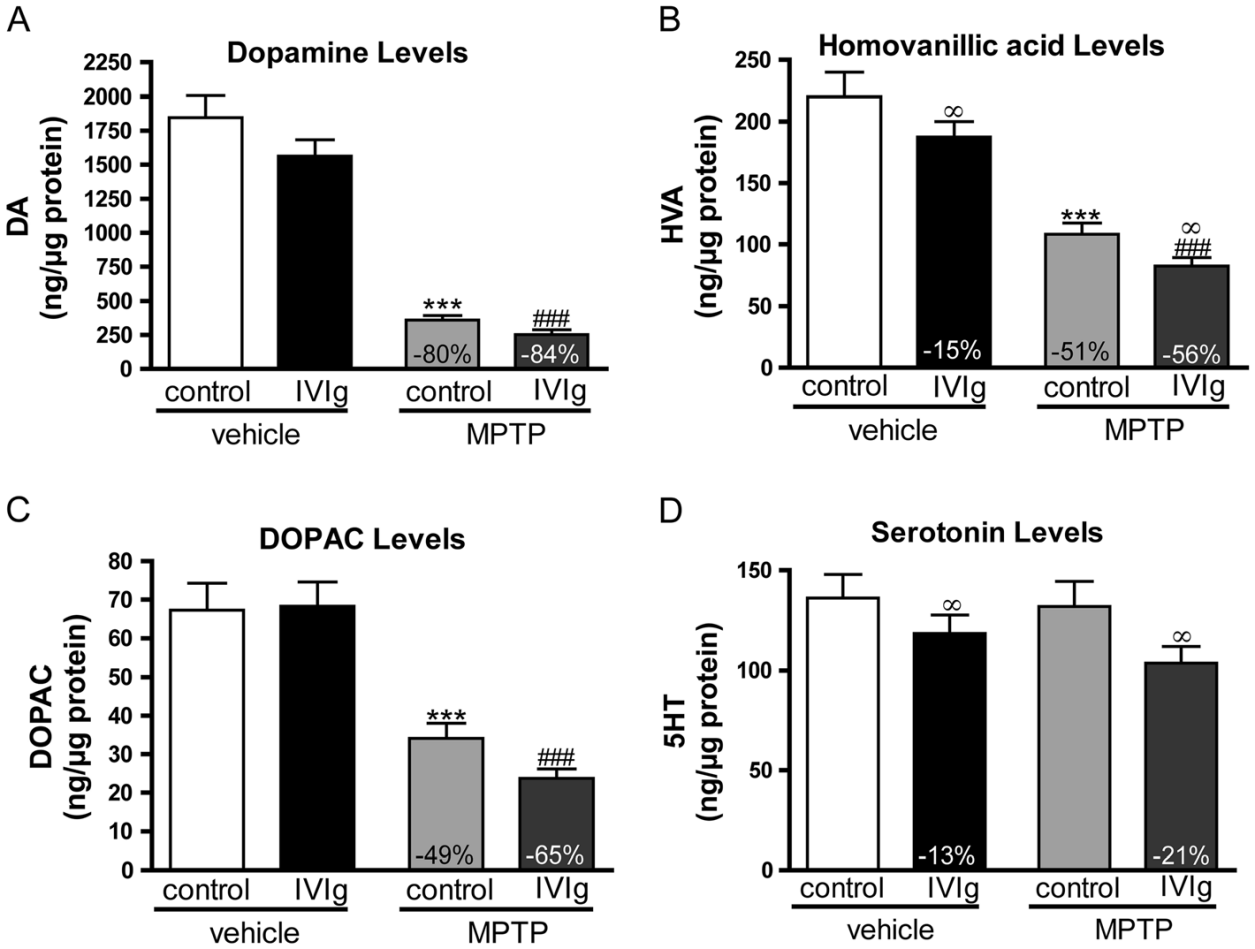
**Lack of beneficial effects of intravenous immunoglobulin on depleted levels of dopamine and its metabolites.** Catecholamines and indolamine were quantified using HPLC in the striatum of mice. Concentrations of (**A**) dopamine (DA), (**B**) homovanillic acid (HVA), and (**C**) 3,4-dihydroxyphenylacetic acid (DOPAC) remained decreased following MPTP injections and subsequent intravenous immunoglobulin (IVIg) treatment. (**D**) Concentration of serotonin. (**A**) to (**D**) Percentage of control values is indicated for significant variation (vehicle-control or vehicle-IVIg, respectively). Two-way analysis of variance (ANOVA) also revealed lower (**B**) HVA and (**D**) serotonin levels in IVIg groups. Values represent mean ± standard error of the mean of 11 to 15 animals per group. Statistical analysis: one-way ANOVA followed by Tukey’s multiple comparison test, ***P <0.001 control-MPTP versus control-vehicle, ^###^*P* <0.001 IVIg-MPTP versus IVIg-vehicle; two-way ANOVA analyses, ^∞^*P* <0.05 IVIg-treated versus control-treated animals.

As evaluated by HPLC quantification, MPTP induced significant reductions in striatal concentrations of DA and its metabolites DOPAC and HVA, reaching 80%, 49%, and 51%, respectively, in IVIg-untreated mice (*P* < 0.01, one-way ANOVA; Figure 5). Similarly, 84%, 65%, and 56% decreases of DA, DOPAC and HVA were observed in the striatum of the IVIg-MPTP mice compared with IVIg-vehicle mice (Figure 5A,B,C). When two-way ANOVAs were performed, the IVIg treatment was associated with a significant decrease in HVA and serotonin (Figure 5B,D) compared with control groups. Therefore, catecholamine data point toward the absence of a restorative effect of the IVIg treatment after the MPTP-induced nigrostriatal lesion.

**Figure 6:**
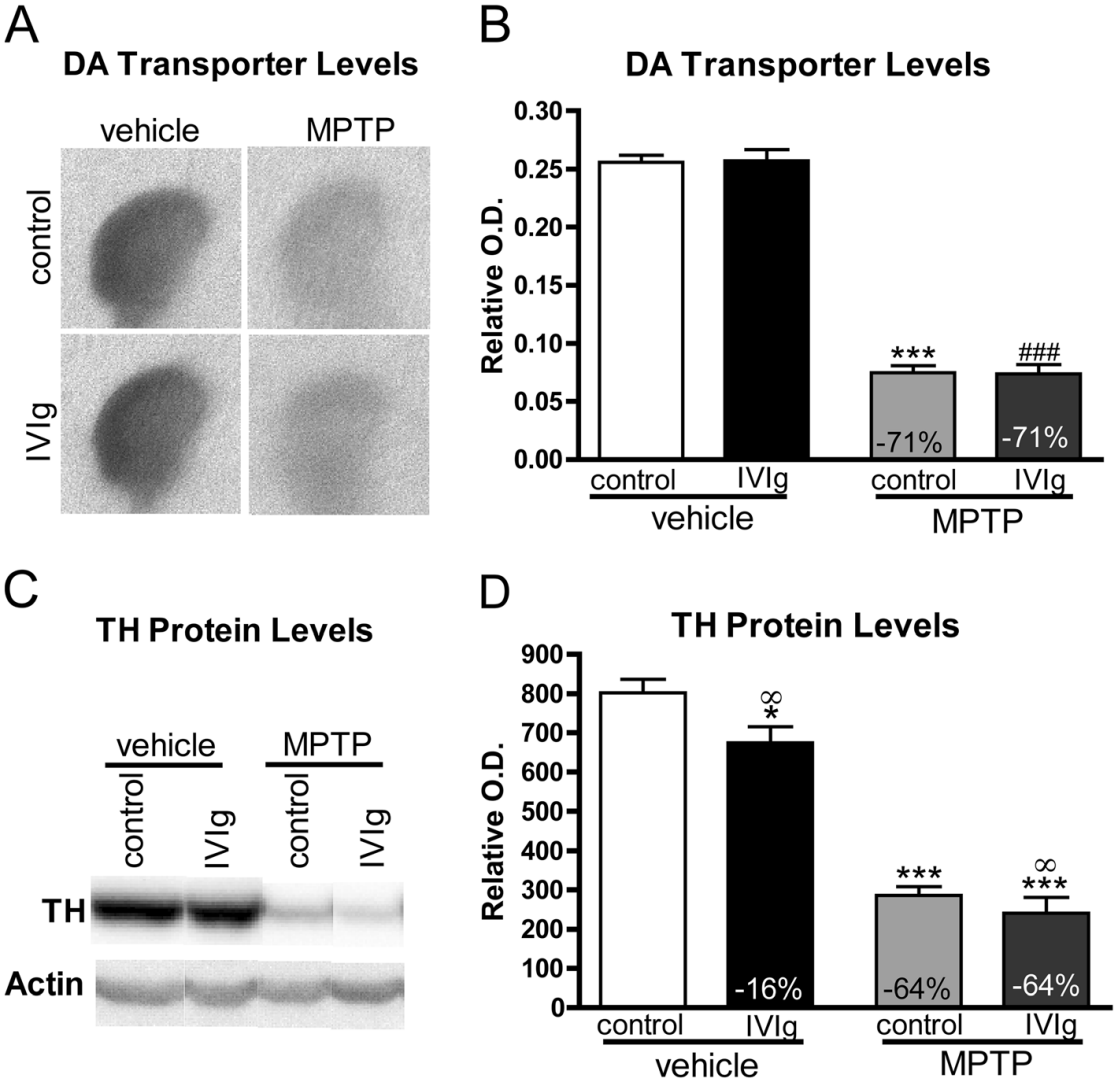
**Decreased tyrosine hydroxylase protein and dopamine transporter levels in the mouse striatum following MPTP treatment**. ^125^I-RTI-121 specific binding to dopamine transporter (DAT) was measured with receptor binding autoradiography and showed a remarkable decrease following MPTP treatment without any effect of intravenous immunoglobulin (IVIg). (**A**) Representative autoradiograms and (**B**) quantification. Statistical analysis: mean ± standard error of the mean (SEM; *n* = 11 to 15/group). One-way analysis of variance (ANOVA) followed by Tukey’s multiple comparison test, ***P <0.001 control-MPTP versus control-vehicle, ^###^*P* <0.001 IVIg-MPTP versus IVIg-vehicle. Striatal tyrosine hydroxylase (TH) protein level was also decreased following MPTP intoxication. (**C**) Representative Western blot of TH in MPTP-mice treated with IVIg or vehicle. (**D**) Densitometric quantification of the Western blots. Data presented as mean ± SEM (*n* = 11 to 15/group). Statistical analysis: one-way ANOVA followed by Dunnett’s multiple comparison test. ***P <0.001, *P <0.05 versus control-vehicle. Besides the downregulating effect of MPTP, a significant IVIg-induced decrease of TH in the striatum of mice was observed in vehicle-treated mice (*P <0.05 vs. control-vehicle) and was further evidenced using two-way ANOVA analyses in both vehicle and MPTP groups (^∞^*P* <0.05 IVIg vs. control). Percentage of control values is indicated for significant variation (vehicle-control vs. MPTP-control or vehicle-IVIg vs. MPTP-IVIg, respectively).

A comparable 71% decrease of ^125^RTI-121-specific DAT binding in both controls and IVIg-treated MPTP mice compared with vehicle mice was measured in the striatum (Figure 6A,B), further supporting the lack of beneficial effects of IVIg. Moreover, TH, the rate-limiting enzyme in the catecholamine synthesis [62], was quantified in the striatum using Western immunoblot analysis. MPTP markedly depleted TH protein levels by 64% in both the MPTP-IVIg and MPTP-control groups compared with their respective controls (Figure 6C,D). IVIg treatment also led to a 16% decrease in TH protein levels in animals not exposed to MPTP. Two-way ANOVAs further underscored a significant decrease of striatal TH protein levels in IVIg-treated groups (vehicle and MPTP) as compared with controls (Figure 6C,D).

### Effects of MPTP and IVIg on nigral dopaminergic neuronal loss

**Figure 7:**
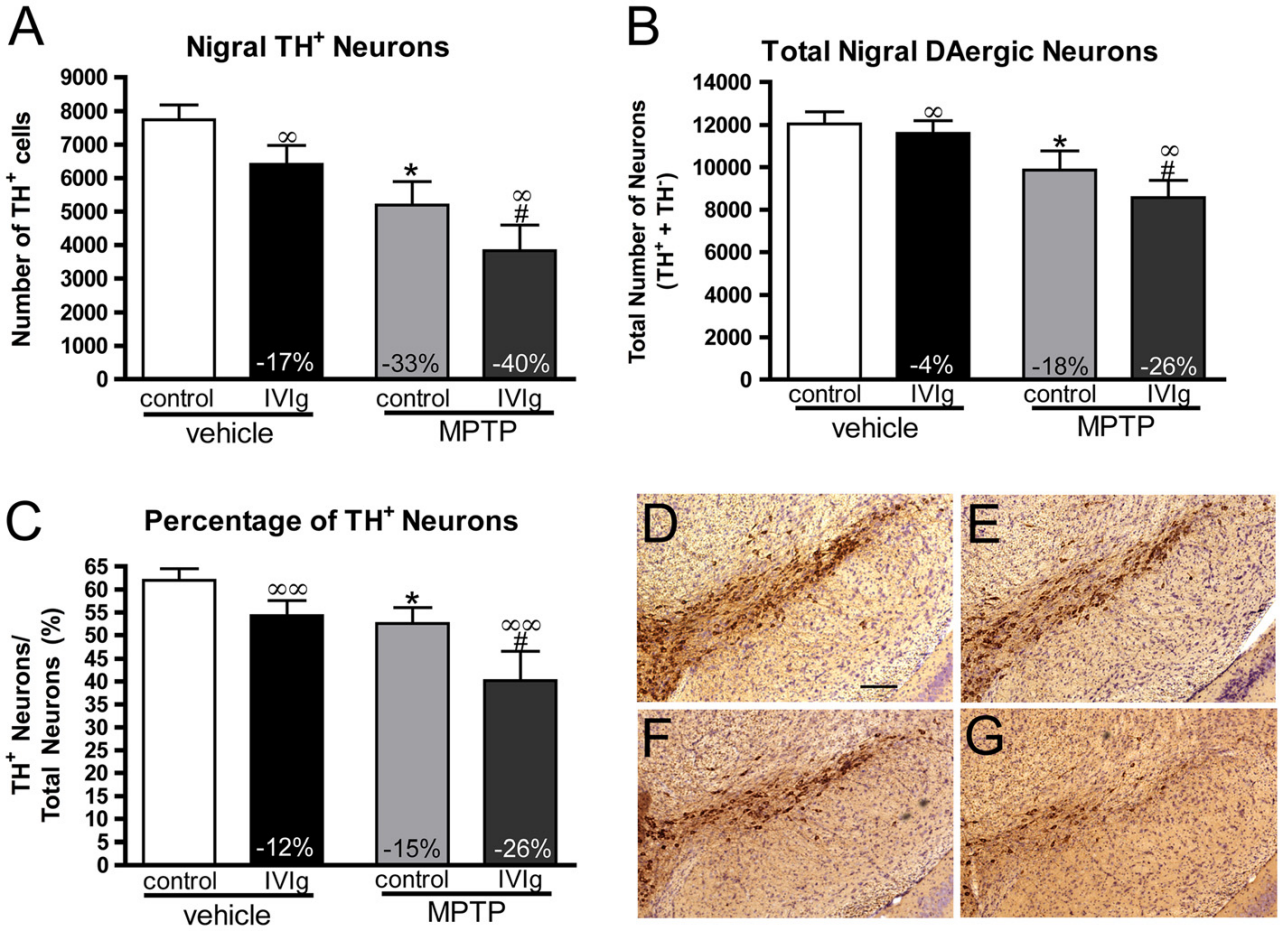
**Quantification of nigral tyrosine hydroxylase-positive neurons following MPTP and intravenous immunoglobulin treatment.** Stereological counts of (**A**) tyrosine hydroxylase (TH)-positive dopaminergic (DAergic) neurons and (**B**) total DAergic neurons of the substantia nigra pars compacta (SNpc) confirming that exposure to MPTP led to a significant nigral neurodegeneration. (**C**) Ratio of TH-positive versus total SNpc neurons in the SNpc (*n* = 12 to 15 animals per group). (**D**) to (**G**) Low power photomicrograph representative of the state of the nigral DAergic system following MPTP and intravenous immunoglobulin (IVIg) treatments. Scale bar: 150 μm. (**D**) Vehicle-control, (**E**) vehicle-IVIg, (**F**) MPTP-control, (**G**) MPTP-IVIg. Statistical analysis: one-way analysis of variance (ANOVA) followed by Tukey’s multiple comparison test, *P <0.05 control-MPTP versus control-vehicle, ^#^*P* <0.05 IVIg-MPTP versus IVIg-vehicle. IVIg treatment (**E**), (**G**) resulted in a diminution in the number of TH-positive neurons compared with vehicle-treated animals (**D**), (**F**), according to two-way ANOVA analyses (^∞^*P* <0.05; ^∞∞^*P* <0.01 IVIg vs. control). Percentage of control values is indicated for significant variation (vehicle-control or vehicle-IVIg, respectively).

As expected, MPTP injections led to a significant decrease in the number of TH-positive DAergic neurons in the SNpc (Figure 7), as determined by immunohistochemistry. Stereological count of TH-positive and cresyl-violet-stained neurons in SNpc revealed a 33% reduction of TH-positive neurons in the MPTP-control group, whereas there was a 40% decrease in the MPTP-IVIg group (Figure 7A), as compared with their respective controls. The total number of SNpc neurons (TH-positive and cresyl-violet-stained) was also decreased by MPTP treatment (Figure 7B). To verify whether IVIg treatment affected the proportion of TH-positive neurons, we measured the ratio of TH-positive neurons versus total SNpc cells, as identified with TH immunohistochemistry and cresyl-violet staining (Figure 7C). Additionally, two-way ANOVA analyses revealed that IVIg treatment led to significant reductions in TH-positive neurons, total number of SNpc neurons and the ratio of TH-positive versus total SNpc neurons in mice.

## Discussion

Our data clearly show that IVIg treatment has an impact on various immune parameters in mice, confirming the immunomodulatory action of IVIg in the periphery. Indeed, systemic administration of IVIg led to the presence of human IgG at the surface of circulating leukocytes, induced a significant decrease in the CD4^+^/CD8^+^ T-cell ratio and increased the Treg percentage. In the present study, we have also assessed the state of the brain DAergic system using a combination of validated markers. However, our results suggest that immunomodulating treatment with IVIg did not translate into neurorestoration of the denervated nigrostriatal DAergic pathway after an acute MPTP insult. Our observations rather suggest potentially negative consequences of IVIg treatment on certain components of the DAergic system, as well as on the health status of the treated animals. The vast majority of preclinical studies aiming to test new compounds for PD are tested in animal models *prior* to the injury (for example, MPTP treatment), thereby probing the neuroprotective properties of the potential therapeutic agents. We opted instead for a neurorestoration study design, in which the injections of IVIg began 20 hours *after* the last MPTP injection. MPTP-induced neurodegeneration is still ongoing at that time, as DAergic denervation stabilizes approximately 7 days after initial MPTP insult [63-65]. Nevertheless, such post-MPTP treatment paradigm is more compatible with an eventual clinical use of IVIg in human PD, which would occur after the diagnosis, when neurodegeneration processes are already engaged [66].

Treg cell adoptive transfer has been previously reported to protect from MPTP-induced nigrostriatal denervation [57-59] in acute MPTP mouse models. In these studies, the amount of Tregs needed to achieve neurorestoration using adoptive transfer ranged between 0.5×10^6^ and 3.5×10^6^ injected into the tail vein 12 hours following the last MPTP injection [57-59]. We also observed a rise in Treg percentage among the CD4^+^ population, reaching up to 9% in the spleen of IVIg-vehicle mice, after a 14-day treatment. However, this increase in Tregs following IVIg administration did not reach the 16 to 20% CD4^+^ Treg proportion previously reported [53,54]. Nevertheless, despite the significant rise in peripheral Tregs, IVIg treatment did not translate into measurable neurorestorative effects. The lack of beneficial effects could be explained by the fact that the rise in Tregs following the initiation of IVIg treatment might have been too slow to allow a sufficient exposure to Tregs to produce any benefits. We also observed a significant increase of Treg percentage after MPTP administration with no additive effects of IVIg. This is in accordance with Rosenkranz and colleagues, who reported a higher suppressive activity of Tregs in PD and AD patients and an increased Treg number associated with aging [67]. Finally, Ramakrishna and colleagues associated the long-term regulation of CNS inflammatory responses to the induction of iCOS^+^CD4^+^ T cells [54], which were left unchanged after the present IVIg treatment. The absence of neurorestorative effects of IVIg could thus also be explained by the lack of expansion of the iCOS^+^CD4^+^ T cells or the Treg population in the MPTP-treated groups.

Injections of IVIg resulted in a mild but significant decrease in the CD4^+^/CD8^+^ T-cell ratio. Such decreases are also observed in IVIg-treated patients [68], suggesting it may be a clinically relevant index of IVIg efficacy. Interestingly, a significant decrease in CD4^+^/CD8^+^ ratio is observed in PD patients as well [69-71], possibly accounted for by an increased susceptibility to apoptosis observed in CD4^+^ T cells, consequent of Fas overexpression [72]. IVIg has been reported to modulate the level of expression of Fas and FasL and to inhibit FasL-dependent apoptosis, in both *in vivo* and *in vitro* studies [34,73-75]. This action of IVIg on the Fas/FasL pathway could have been translated into neurorestoration. However, while being consistent with an immunoregulatory action, the effect of IVIg on the CD4^+^/CD8^+^ T-cell ratio was not associated with beneficial post-MPTP outcomes on various DAergic markers.

The lack of efficacy of IVIg may also reflect the poor CNS access owing to the presence of the blood–brain barrier. However, our data rather suggest that IVIg displayed significant central bioavailability after systemic administration. Indeed, a fraction of intraperitoneally administered IVIg was detected in the striatum of treated mice using a specific ELISA, consistent with a previous report where peripherally administered IVIg was also detected in APP/PS1 mouse brain using immunohistochemistry [76]. A number of studies have reported data consistent with the penetration of a fraction of systemically administered antibodies into brain tissues leading to central therapeutic effect [38,77,78]. Interaction between Fc gamma receptor (FcγR) and immunoglobulins is essential for the initiation of cellular and humoral responses. In the CNS, FcγR are expressed on endothelial cells, neurons, microglia, oligodendrocytes and astrocytes (as reviewed in [79]) and the IVIg migration to critical regions of the brain, such as the striatum and SNpc in PD, might act as a central immunomodulating agent. A previous report showed that approximately 30% of pigmented SNpc neurons were IgG-positive [80] in PD patients but not in controls. This suggests that IgG can access the brain during the course of the disease. However, we found no increase in striatal IgG content in MPTP-treated animals. The amount of human IgG detected in the brain of treated mice suggests that low central bioavailability is unlikely to be the sole reason for the lack of efficacy of IVIg in restoring the DAergic pathways.

After systemic injection, MPTP produces a reproducible lesion of the nigrostriatal DAergic pathway by causing oxidative stress, mitochondrial damage and neuronal cell death, as in idiopathic PD. Validation of disease-modifying treatments before clinical trial initiation is therefore often performed in MPTP-treated rodent models [81,82]. However, these models are not without important limitations [83]. First, the MPTP model used here does not generate a massive degeneration (−30 to 40% of TH-positive cells), which is required for clinically detectable motor symptoms in humans (−50 to 60%) [84,85]. This explains, at least in part, why motor symptoms in the MPTP mouse model are insufficiently reliable for systematic assessment [83,86-89] and were not evaluated here after IVIg treatment. To investigate the symptomatic effects of IVIg, the use of the more expensive MPTP monkey model should be considered instead [83,90]. Second, the acute mouse MPTP model does not replicate α-synucleinopathy or Lewy bodies, which are pathognomonic of PD [91-93]. The use of other models such as the chronic infusion MPTP models or transgenic mice overexpressing human α-syn might be helpful for these purposes [81,93-95]. Third, the response of a mouse model to human IVIg may differ from humans. Indeed, the absence of positive outcome in our study might be the result of inadequate interactions between human IgG and mouse FcγR, a hypothesis only testable with the use of mouse IgG. However, given that over 2,500 mice would have been required to generate the ~5 g IgG used in this study, murine IgG is scarcely used in preclinical investigations. In a passive model of idiopathic thrombocytopenic purpura, human IVIg and purified mouse IgG shared the same kinetics to restore platelet counts, thus validating the use of human IVIg to study human therapy in mouse models [96]. Since monomeric human IVIg is well tolerated in mice, mouse models of numerous diseases are now routinely used to investigate its efficacy as well as its mechanisms of action [35,53,97,98].

The unexpected deleterious effect of IVIg on TH expression is an intriguing observation that is particularly challenging to explain. On the one hand, a plethora of compounds such as nicotine, cannabinoid agonists and progesterone receptor isoforms [99-101] have been shown to modulate TH expression without obvious harmful effects on the DAergic system. Similarly, our data suggest that IVIg regulates TH expression at the protein or RNA levels. On the other hand, the observed decrease in striatal TH protein levels associated with a trend toward decreased catecholamines, serotonin, nigral TH-positive and total neurons can also be interpreted as a deleterious effect of IVIg on the murine DAergic system. Although acute MPTP administration does not lead to α-syn-positive nigral inclusions [91-93], α-syn-deficient transgenic mouse models are more resistant to MPTP, suggesting a possible implication of α-syn in the MPTP toxicity [102-106]. Increased autoantibodies to α-syn are present in the sera of PD patients [107,108], and stereotactic injection of human IgG purified from the sera of PD patients into mice SNpc produces a FcγR-dependent microglial activation and a 40% TH-positive cell loss in the SNpc [109]. Since natural anti-α-syn antibodies in IVIg preparations have been recently identified [40], it is tempting to speculate that IVIg could have modulated the nigrostriatal toxicity of MPTP by binding to α-syn.

## Conclusion

Despite the fact that current knowledge of IVIg mechanisms of action holds promising characteristics for the treatment of PD, our results do not provide evidence of a neurorestorative effect of IVIg treatment on the nigrostriatal system of the MPTP-treated mouse. Our data on the general health status, DAergic cell count, TH protein levels and HVA striatal concentrations all suggest that IVIg not only failed to generate beneficial effects, but had a slight detrimental impact on the DAergic system. Such possible harmful consequences flag the need to proceed with caution before initiating clinical trials in PD patients.

## Abbreviations

AD: Alzheimer disease; ANOVA: analysis of variance; BSA: bovine serum albumin; CD: cluster of differentiation; CNS: central nervous system; DA: dopamine; DAergic: dopaminergic; DAT: dopamine transporter; DOPAC: 3,4-dihydroxyphenylacetic acid; ELISA: enzyme-linked immunosorbent assay; FasL: Fas ligand; FcγR: Fc gamma receptor; FITC: fluorescein isothiocyanate; HPLC: high-performance liquid chromatography; HVA: homovanillic acid; IFN: interferon; IL: interleukin; I-RTI-121: 3β-(4-^125^I-iodophenyl) tropane-2-carboxylic acid isopropylester; IVIg: intravenous immunoglobulin; MPTP: 1-methyl-4-phenyl-1,2,3,6-tetrahydropyridine; PBS: phosphate-buffered saline; PD: Parkinson’s disease; PE: R-phycoerythrin; TGFb: transforming growth factor-beta; TH: tyrosine hydroxylase; TNF: tumor necrosis factor; Treg: regulatory T cell; SNpc: substantia nigra pars compacta; α-syn: α-synuclein.

## Competing interests

FCa and RB have received funding from Grifols for other research projects on IVIg. The remaining authors declare that they have no competing interests.

## Authors’ contributions

IS-A participated in the design of the experiments, performed the animal studies and most of the postmortem analyses, analyzed the data and wrote the manuscript. MB participated in the design of the experiments and animal studies, executed the HPLC analyses and revised the manuscript. IP performed the flow cytometry and ELISPOT experiments. JD-O participated in animal studies, carried out the stereological quantification and revised the manuscript. FCi provided scientific input and revised the manuscript. RB provided resources for flow cytometry analyses and revised the manuscript. FCa conceived and designed the study, analyzed the data and wrote the manuscript. All authors read and approved the final version of the manuscript.
